# Supplementary material for: The late formation of chondrites as a consequence of Jupiter-induced gaps and rings
Source: Sci Adv. 2025 Oct 22;11(43):eady4823. doi: 10.1126/sciadv.ady4823 (PMC12542937; doi:10.1126/sciadv.ady4823)
Supplement: Supplementary file 1 — Supplementary Text Figs. S1 to S6 [file sciadv.ady4823_sm.pdf]

Science Advances
AAAS

Supplementary Materials for

The late formation of chondrites as a consequence of Jupiter-induced gaps and rings

Baibhav Srivastava and André Izidoro

Corresponding author: Baibhav Srivastava, baibhav.s@rice.edu; André Izidoro, izidoro@rice.edu

Sci. Adv. **11**, eady4823 (2025)
DOI: 10.1126/sciadv.ady4823

This PDF file includes:

Supplementary Text
Figs. S1 to S6

Supplementary Text

The effects of viscosity

The disk viscosity plays an important role in the formation and location of pressure bumps (rings in the gas distribution) in the disk (*46*). A higher disk viscosity results in greater damping of the spiral waves generated by the planet as they propagate, which in turn limits the number of pressure bumps that can form. Conversely, a low-viscosity disk allows the spiral arms to propagate farther from the planet, leading to shocks in the disk and the formation of multiple pressure bumps (*46, 47*). In figure S1, we see the evolution of surface densities for three different disk α viscosities: 10^{-3}, 10^{-4}, and 10^{-5}. These simulations are similar to the nominal one but utilize closed boundary conditions instead. As the viscosity decreases, pressure bumps are observed to form farther from the planet. Note that the observed differences in bump amplitudes in Figure 2 and figure S1 arise from differences in the simulation time and gas surface density (due to different boundary conditions). More importantly, figure S1 confirms that the five bumps seen in Figure 2 of the main paper are not artifacts caused by specific boundary conditions. As stated in the main paper, our scenario favors a low-viscosity regime for the Sun's natal disk, specifically $\alpha \lesssim 10^{-4}$.

Supplementary figure S2 shows the potential vorticity and the normalized surface density at 200 and 3700 orbits in the simulation of Figure 2 (main paper). As seen, the locations where pressure bumps form correspond to specific locations where the gas shocks, as indicated by the disk potential vorticity. A high normalized potential vorticity indicates the location of a gap.

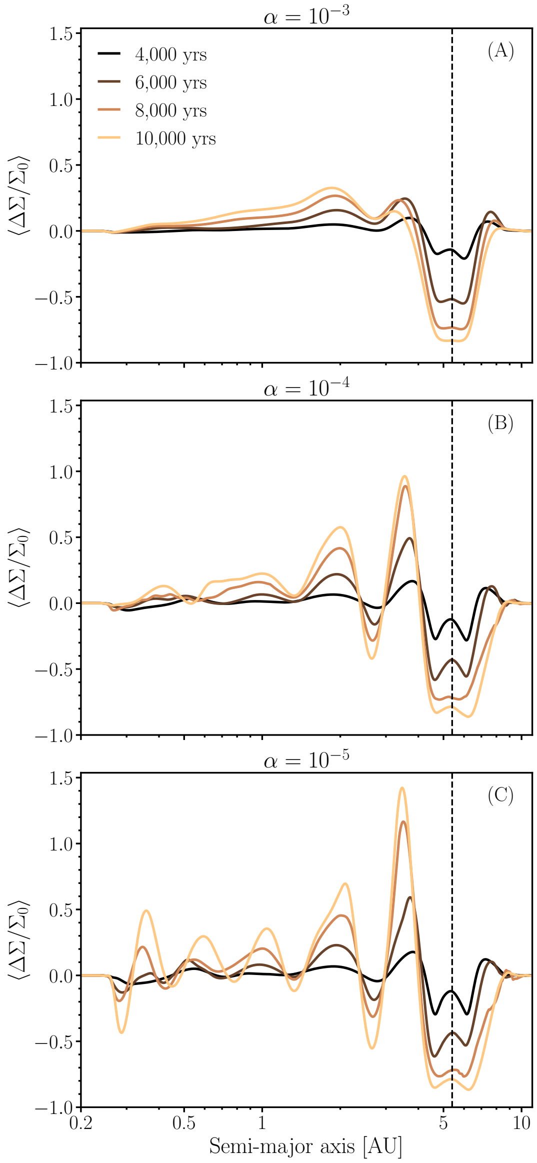

Figure S1: **Snapshots of normalized gas surface density in hydrodynamical simulations with a Jupiter-mass planet placed at 5.4 au in disks with different α-viscosity parameters.** Similar to our nominal simulations (e.g., Figure 2), these simulations also start from a power-law disk profile following the MMSN model, but we instead use closed boundary conditions with damping. Jupiter is kept in a non-migrating orbit, as indicated by the dashed line. The three panels illustrate the evolution of normalized surface density for disks with different viscosities: $\alpha = 10^{-3}$ (A), $\alpha = 10^{-4}$ (B), and $\alpha = 10^{-5}$ (C). These simulations were performed with a resolution of 24 cells per scale height at 1 au.

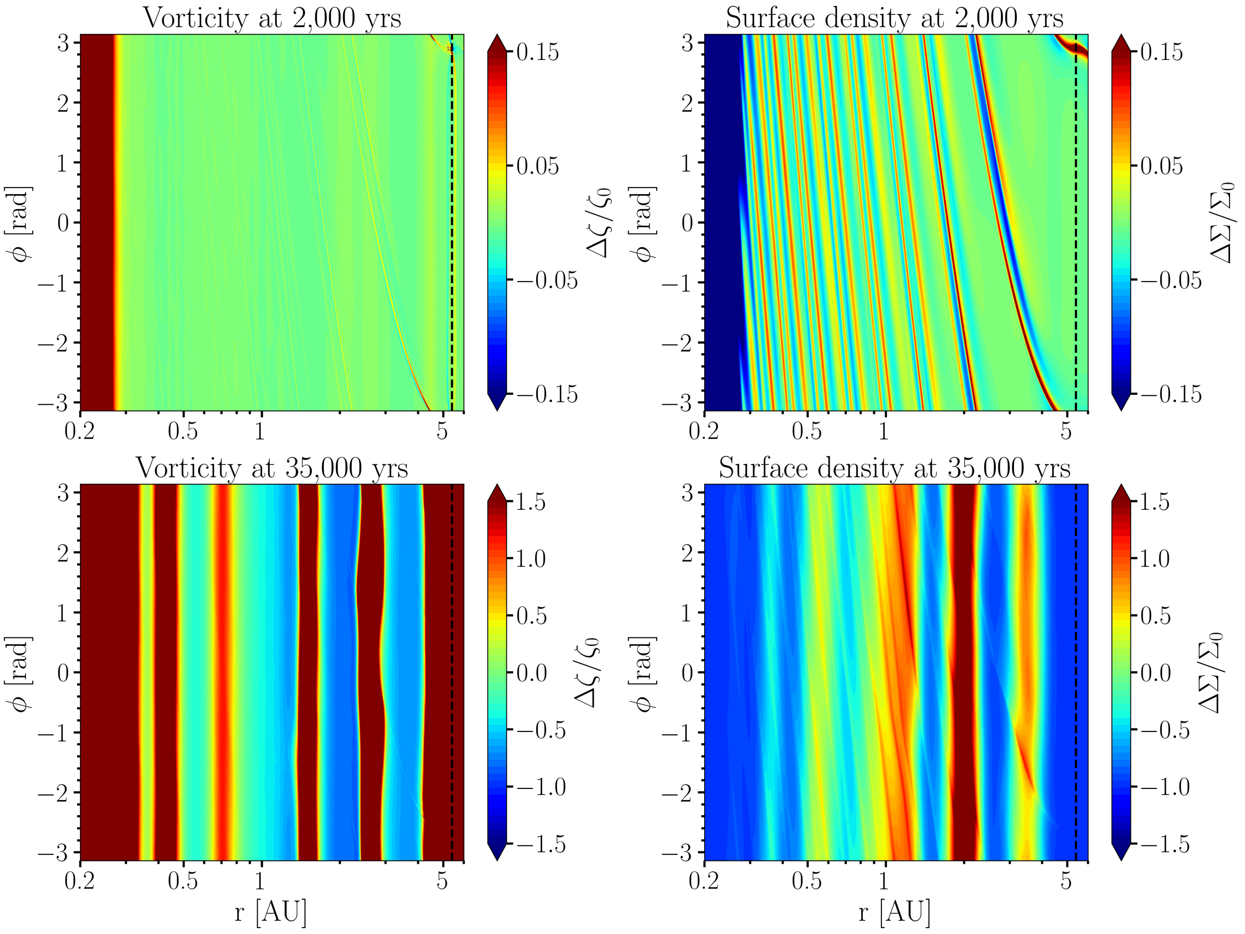

Figure S2: **Normalized potential vorticity and surface density of the disk at 2,000 and 35,000 years of our nominal simulation.** This simulation is the same as that shown in Figure 2 of the main paper. The left panels display the disk's potential vorticity, while the right panels show the surface density. The top and bottom rows correspond to different evolutionary times: 2,000 years (top) and 35,000 years (bottom). The x-axis represents the radial direction, and the y-axis corresponds to the azimuthal direction. Both vorticity and surface density are normalized to their initial values to enhance the visibility of fine structures.

Dust production throughout the inner disk

In our nominal simulations, we assume that during the growth of terrestrial planets through planetesimal accretion, dust is produced exclusively between 0.7 and 2 au – a region largely overlapping with the zone where terrestrial planetary embryos are forming (see our nominal simulations and (*26, 70*)). In figure S3, however, we adopt a more generous distribution, initializing and adding dust through-

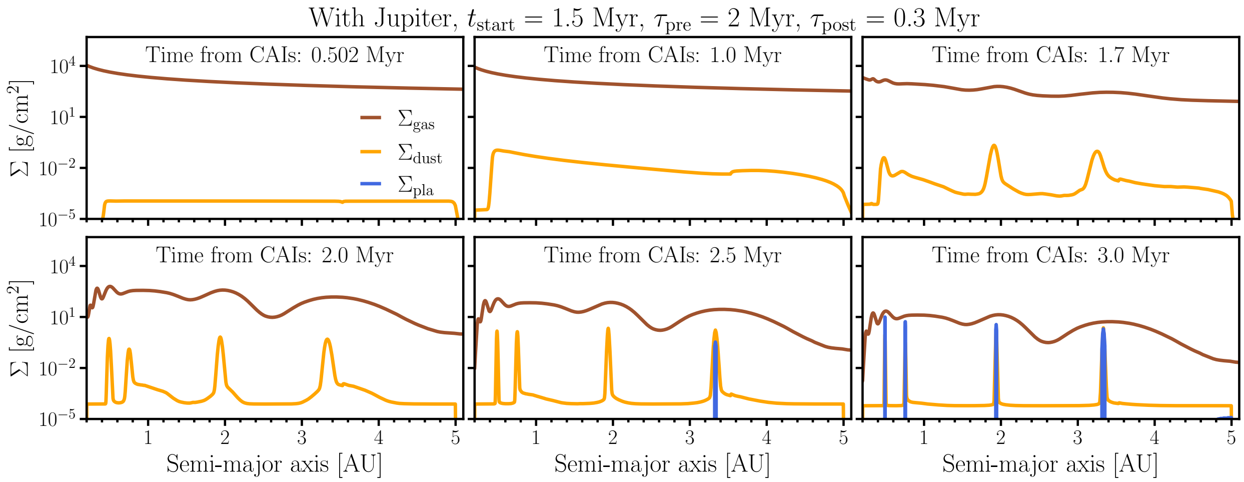

Figure S3: **Snapshots of surface densities of gas, dust, and planetesimals from a dust advection-diffusion simulation incorporating planetesimal formation, with dust production throughout the inner disk** The setup for this simulation is similar to that shown in Figure 5, with the key difference being that dust is initially introduced into a wider region, extending from 0.2 to 5 au. Jupiter begins to form at t_{start} = 1.5 Myr, creating pressure bumps and accelerating the inner gas disk depletion timescale to 0.3 Myr. Pressure bumps trap dust and ultimately lead to the formation of planetesimals in four distinct reservoirs.

out the entire inner disk. In Supplementary figure S3, dust is trapped in four out of five pressure bumps in the inner disk, ultimately leading to planetesimal formation in all of them.

Growth-tracks for planetary embryos with 2 Myr gas disk depletion timescale

To evaluate the impact of the gas disk depletion timescale on the growth and migration of planetary embryos, we performed N-body simulations similar to those discussed in the main text but with a fixed gas disk depletion timescale of 2 Myr throughout the simulation. The results of these simulations are presented in Supplementary figure S4.

Our results show that even without the relatively rapid gas depletion observed in our nominal simulations (e.g. τ_{post} = 0.3 Myr) following Jupiter's formation, the evolution of terrestrial planet embryos remains qualitatively unchanged when Jupiter forms within 1.5 Myr. The migration of growing planetary embryos is slowed down by planet traps created by Jupiter, leading to the accumulation of planetary embryos, primarily around 0.8 au.

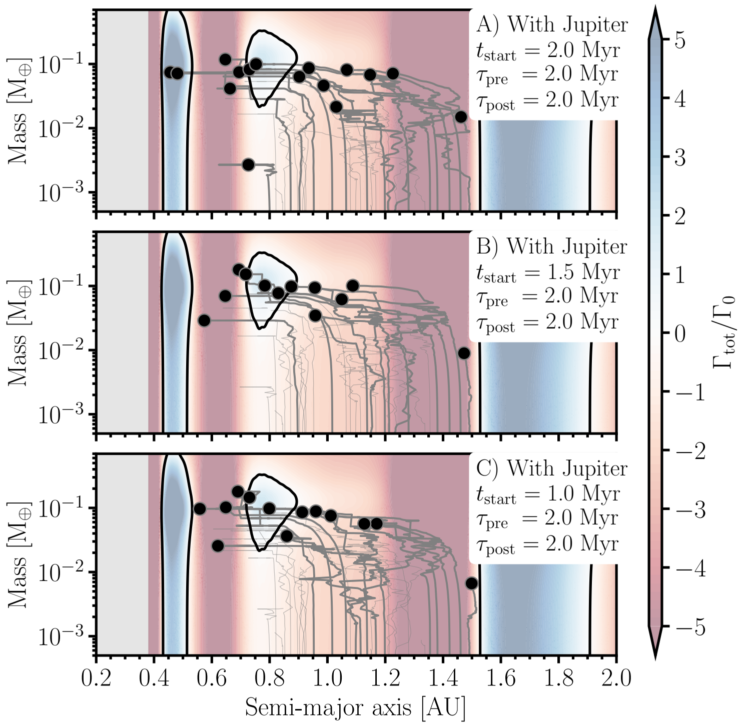

Figure S4: **Growth-migration tracks of planetary embryos in simulations with Jupiter forming at varying times and with 2 Myr depletion timescale for gas density** The setup of the simulation is similar to the nominal simulations presented in Figure 4. However, here, the gas disk depletion timescale remains fixed at 2 Myr during the entire course of the simulation and does not shorten after Jupiter's formation ($\tau_{\rm pre} = \tau_{\rm post} = 2$ Myr). The three panels show three different scenarios for the timing of Jupiter's formation. From top to bottom, panels A), B), and C) show simulations in which Jupiter begins to grow at 2, 1.5, and 1 Myr, respectively.

Effects of gas depletion on the formation of late planetesimals

In the main paper, we investigated the role of accelerated gas depletion in the formation of late-stage planetesimals. To further illustrate its importance, we present the results of one of our dust diffusion-advection simulations that neglects the accelerated gas disk depletion induced by Jupiter. In this scenario, Jupiter forms at 1.5 Myr and induces pressure bumps in the disk, but the gas disk depletes at a constant timescale of 2.0 Myr throughout the simulation. The result of this simulation is shown in figure S5.

As in the nominal simulation, dust begins to accumulate in the pressure bumps after 1.5 Myr.

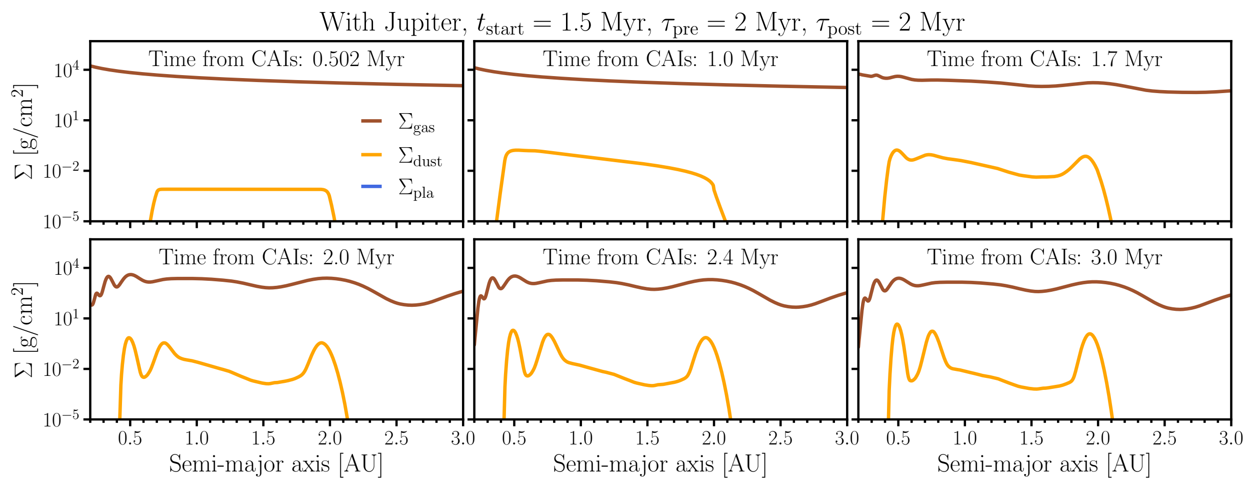

Figure S5: **Snapshots of surface densities of gas, dust, and planetesimals from a dust advection-diffusion simulation incorporating planetesimal formation with 2 Myr depletion timescale for gas density** The setup of this simulation is the same as shown in Figure 5, with one key difference being that the disk dissipation timescale does not change after the formation of Jupiter, specifically $\tau_{pre} = \tau_{post} = 2$ Myr. As Jupiter begins to form at 1.5 Myr, pressure bumps lead to the accumulation of dust. However, planetesimal formation does not occur in the disk, as the gas densities remain too high and planetesimal formation conditions are not met.

However, due to the slower gas depletion, the local dust-to-gas ratio never exceeds unity, preventing the formation of planetesimals within the first 3 Myr. These findings indicate that both the presence of pressure bumps and the accelerated depletion of the gas disk are crucial for the formation of the second generation of planetesimals.

Inner and outer gas disk

Due to the adopted open boundary conditions in our hydrodynamical simulations, the total gas mass in both the inner and outer disk decreases over time as gas is lost through the inner and outer edges. Supplementary figure S6 illustrates the evolution of the disk mass in our nominal simulation. The blue curve represents the mass obtained from the FARGO3D simulations, while the orange curve shows the mass calculated from the instantaneous mass accretion rates at the respective disk edges

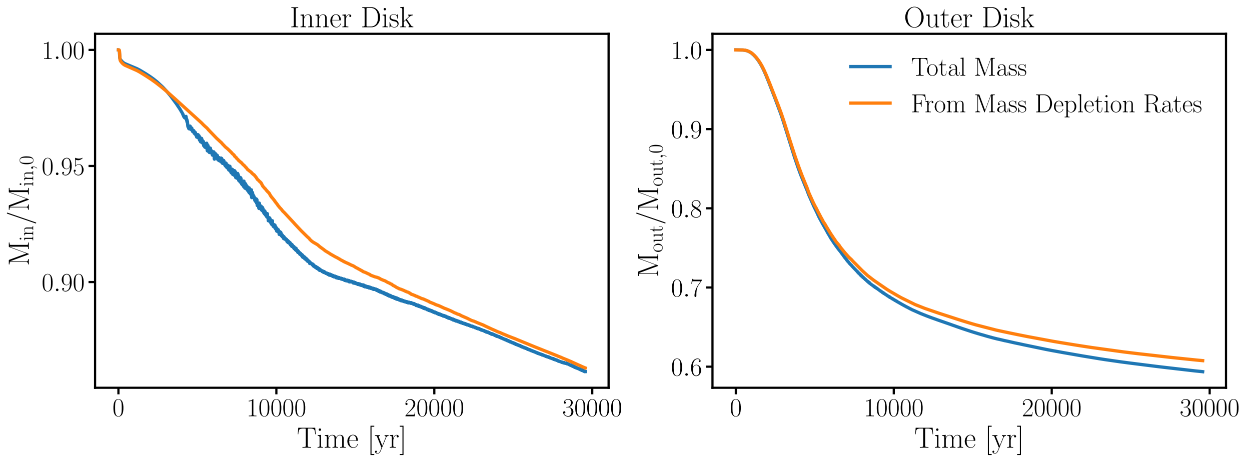

Figure S6: **Evolution of the disk mass in the inner (left) and outer disks (right) as a function of time in our nominal hydrodynamical simulation, including Jupiter.** The mass is normalized by the initial mass in the respective region. The inner region is defined as the region where $r < 5.4$ au and the outer disk as $r > 5.4$ au. The blue curve is the same as the blue curve in Figure 3, while the orange curve represents the mass in each region when integrating the accretion rates at the inner and outer boundaries of the disk. The main purpose of this figure is to show that the inner and outer disks are efficiently disconnected by Jupiter.

using the following expression:

$$M_X(t) = M_{X,0} - \int_0^t \dot{M}X(t)dt = M_0 - \int_0^t \int_X \Sigma_{\rm gas}(r,t)v_r(r,t)drdt, \tag{S1}$$

where X denotes either the inner or outer disk region. The negligible difference (within a few percent) between the two curves indicates that the mass loss from these regions is primarily due to outflows at the disk boundaries, with minimal mass exchange between them. This shows that Jupiter acts as an effective barrier between the inner and outer solar system.
